# Editorial Notes

**Published:** 1936

**Authors:** 


### Editorial Notes

---

**New President of the Queen Victoria Jubilee Convalescent Home.**

Mr. Robert Hall Warren was unanimously elected President of the Convalescent Home at a Governors' Meeting held on 28th February, 1936. Mr. Warren, who has been Honorary Treasurer to the Home for a number of years, has consented to continue to discharge the duties of that office in addition to holding the Presidency.

**Graded Milk.**

We welcome the new milk designations that are to come into force shortly. [Draft Milk (Special Designations) Order, 1936.] After the date designated milk will all be sold under licence as follows :—

| RAW. | PASTEURIZED. |
|---|---|
| 1. Tuberculin tested. | 1. Certified (pasteurized) tuberculin tested. |
| 2. Accredited (not tuberculin tested). | 2. Pasteurized (any milk): not tuberculin tested. |

The term " Grade A " disappears.

The *raw* milks are to be produced, as well as retailed, under licence. In addition to rigorous inspection they must pass a specific methylene blue reduction test. The milk is to be delivered in sealed containers or bottles. The cap must contain the name and address of the bottler and may state day and time of production.

The *pasteurized* milks are standardized by bacterial contents. The certified grade is to be produced under licence, and must be delivered in capped bottles or sealed containers. Pasteurized non-tuberculin tested need not be produced under licence or sold in bottles, but all vessels in which the milk is transported or is exposed or offered for sale shall be labelled " Pasteurized Milk."

The raw milks, so long as they conform to standard, have the economic advantages of early delivery and longer keeping qualities. Their main disadvantage is the necessity for home pasteurization, or other form of heat, if possible infections by B. abortus, streptococci and extraneous pathogens are to be avoided. In other words, they are not " safe " milks from the medical standpoint, even if "Tuberculin tested" or "Accredited."

The grade of certified (pasteurized) milk has, however, provided us with a really *safe*, clean milk, and that of pasteurized with a safe and reasonably clean one. Had the order directed the labelled dating of these milks, as it does for raw milks, the housewife's objection that pasteurized milk does not keep sweet would have been almost overcome. At all events, it would have tended to ensure earlier delivery. The time taken at present for collection, transport, processing and delivery calls for reduction. Much of the pasteurized milk now available is at least thirty-six to forty-eight hours old before it reaches the consumer.

More important, however, is the necessity for assuring the consumer that pasteurization is consistently carried out at maximum efficiency. This is a responsibility that local authorities have to bear. Probably as time goes on public opinion will compel every urban and city authority to ensure that all milk offered for sale has been efficiently pasteurized. It is satisfactory to state that in the Bristol area the several plants are closely controlled by regular inspection and laboratory examinations.

Since the Draft Milk (Special Designations) Order, 1936, was made public the Minister of Health has received a deputation from the Certified and Grade "A" (Tuberculin Tested) Milk Producers' Association. This Association expressed to the Minister regret that the Draft Order did not maintain a separate designation for the highest grade of raw milk, and, more particularly, that it permitted the use of the designation "Certified" in connection with Tuberculin Tested milk after pasteurization.

The National Farmers' Union has also interviewed the Minister of Health, raising objections to the Draft Order. The Milk and Dairy Produce Committee of the N.F.U. (the Council of the Union concurring) had taken strong objection to the proposed designation "Certified (Pasteurized)" as apart from the suggestion it conveyed that "Certified" raw milk could be improved by pasteurization, the decision to use the designation "Certified" no longer in relation to raw milk meant that the good name attached to "Certified" was being utilized wholly in favour of pasteurization. It was felt that the use of "Certified" was to be preferred to "Tuberculin Tested" in relation to raw milk.

The Committee were also of the opinion that the proposed date of commencement of the Order, namely April 1st, 1936, did not provide adequate time for the consideration and explanation of the proposals, for the disposal of old caps and bottles and purchase of new ones to conform to new requirements, or for the proper consideration of all the changes involved in the proposed new methods of bacterial examination.

Following on these representations the Minister of Health has announced that the Draft Order will not come into operation on April 1st, and is under reconsideration.

It is disconcerting to find that the Ministries of Health and Agriculture should have framed so important a Draft Order without ascertaining the views of such vitally interested bodies as the Milk Producers' Association and the National Farmers' Union. There is, however, another view-point to be considered, namely the consumers', especially the infant consumers'—they, whose lives (not livelihoods) may well be put in jeopardy, have not, so far as we are aware, approached the Minister of Health with a deputation.

**Colston Medical Research Fellowships.**

THE Council of the Colston Research Society, Bristol, offers one or more Fellowships for 1936–37 of not less than £100 per annum for Research work primarily in Clinical subjects, though other medical subjects are not excluded. The work must be carried out in the University of Bristol or in any of the Clinical Institutions associated with the University.

Awards will be made in June or July, 1936. Applications should be forwarded forthwith to the Hon. Secretary of the Colston Research Society, 12 Small Street, Bristol.

**Joseph Williams, ob. 1849.**

THE Local Government Act of 1929 has had an unforeseen effect in Bristol. The replacement of the Board of Guardians by the City Council and its Committees seems to have severed the traditional connection of the former with the Bristol Corporation of the Poor.

The City Council no longer boasts of a splendid origin in that first Corporation of the Poor to be established in England, and repudiates any responsibility for its actions. This has led to a deplorable neglect of a notable monument erected in 1849 to the memory of a young Bristol doctor who sacrificed his life during the cholera epidemic of that year by his fearless care of the sick of our city. The following copy of the inscription on his tomb at Arno's Vale gives a complete account of his work and untimely death :—

JOSEPH WILLIAMS.

(SURGEON.)

*Died of Cholera. Sept. 8th,* 1849.

*Aged* 26.

The best testimony to his character while living is the brief but heroic narrative of his death.

When the pestilence which had been raging in Bristol broke out in the crowded wards of Stapleton Workhouse, he was among the first to volunteer on a service fraught with peril.

Impelled by an ardent sense of duty, and the philanthropic intrepidity which braves all danger to aid the distressed, he laboured day and night heedless of friendly remonstrance, amid the sick and dying, until strength and nature being overwrought he was seized by the fatal malady from which he was the means of rescuing others, and fell with the calmness of a Christian on one of the noblest causes in which life can be laid down.

The Corporation of the Poor of the City of Bristol have caused this tomb and inscription to be placed above his remains to mark their grateful esteem for the memory of a man to whose skill, courage and devotion the Public were much indebted at a season of great gloom and suffering.

A visit to his tomb is sufficient evidence of the apathy of those who have inherited the mantle and duties of the Corporation of the Poor. But perhaps there are no heritors either to its duties, nor to the spirit by which it was actuated in its great pioneer work, splendidly begun in 1699, and continued ungrudgingly until 1929.

The monument to Joseph Williams is in danger of complete decay. It needs repair urgently. Three years ago the matter was brought to the notice of the City Council, whose reply was that "the Corporation have no power to spend money for this purpose."

The reply, if truly reported, is pitiful.

